# Pre-Conception Maternal Obesity Confers Autism Spectrum Disorder-like Behaviors in Mice Offspring Through Neuroepigenetic Dysregulation

**DOI:** 10.3390/cells14151201

**Published:** 2025-08-05

**Authors:** Nina P. Allan, Amada Torres, Michael J. Corley, Brennan Y. Yamamoto, Chantell Balaan, Yasuhiro Yamauchi, Rafael Peres, Yujia Qin, Vedbar S. Khadka, Youping Deng, Monika A. Ward, Alika K. Maunakea

**Affiliations:** 1 Yanagimachi Institute for Biogenesis Research, Department of Anatomy, Biochemistry and Physiology, John A. Burns School of Medicine, University of Hawaiʻi at Mānoa, Honolulu, HI 96822, USAmicorley@health.ucsd.edu (M.J.C.); brennany@hawaii.edu (B.Y.Y.); yyamauch@hawaii.edu (Y.Y.); peres@hawaii.edu (R.P.); mward@hawaii.edu (M.A.W.); 2 Department of Medicine, Division of Geriatrics, Gerontology and Palliative Care, University of California San Diego, La Jolla, CA 92093, USA; 3 Bioinformatics Core Facility, Department of Quantitative Health Sciences, John A. Burns School of Medicine, University of Hawai’i at Mānoa, Honolulu, HI 96822, USA

**Keywords:** neuroepigenetics, maternal obesity, autism, DNA methylation

## Abstract

Autism spectrum disorder (ASD) is a complex neurodevelopmental condition with early-life origins. Maternal obesity has been associated with increased ASD risk, yet the mechanisms and timing of susceptibility remain unclear. Using a mouse model combining in vitro fertilization (IVF) and embryo transfer, we separated the effects of pre-conception and gestational obesity. We found that maternal high fat diet (HFD) exposure prior to conception alone was sufficient to induce ASD-like behaviors in male offspring—including altered vocalizations, reduced sociability, and increased repetitive grooming—without anxiety-related changes. These phenotypes were absent in female offspring and those exposed only during gestation. Cortical transcriptome analysis revealed dysregulation and isoform shifts in genes implicated in ASD, including *Homer1* and *Zswim6*. Whole-genome bisulfite sequencing of hippocampal tissue showed hypomethylation of an alternative *Homer1* promoter, correlating with increased expression of the short isoform *Homer1a*, which is known to disrupt synaptic scaffolding. This pattern was specific to mice with ASD-like behaviors. Our findings show that pre-conceptional maternal obesity can lead to lasting, isoform-specific transcriptomic and epigenetic changes in the offspring’s brain. These results underscore the importance of maternal health before pregnancy as a critical and modifiable factor in ASD risk.

## 1. Introduction

Autism spectrum disorder (ASD) encompasses a group of complex neurodevelopmental conditions characterized by deficits in social communication, impaired social interactions, and restricted repetitive behaviors [1]. ASD affects approximately one in thirty-six children in the United States, with a male-to-female ratio near 3:1 [2,3]. While the disorder has a heritable component, fewer than 10% of affected individuals carry strongly penetrant genetic mutations, highlighting a significant role for environmental and epigenetic influences during early brain development [4,5,6,7].

Recent studies suggest that maternal metabolic status, particularly obesity, may increase the risk of ASD in offspring [7,8,9,10]. This association aligns with the Developmental Origins of Health and Disease (DOHaD) hypothesis, which posits that early-life environmental exposures—including those occurring prior to or during pregnancy—can modify developmental trajectories and increase disease susceptibility later in life [8,9,11,12,13]. Although epidemiological data link maternal obesity with increased ASD risk, the precise biological mechanisms and timing of susceptibility remain unknown.

Epigenetic regulation is a strong candidate mechanism for mediating gene–environment interactions in neurodevelopment. Genome-wide studies in both ASD mouse models and postmortem human brain tissues from affected individuals have identified widespread transcriptomic and epigenomic dysregulation of genes involved in neuronal differentiation, cortical architecture, and synaptic functions [14,15]. However, it remains unclear whether maternal metabolic insults influence offspring neurodevelopment through pre- or post-implantation mechanisms, or whether they differentially shape the transcriptional and epigenetic landscape in a manner consistent with ASD-related phenotypes. To address this gap, we combined in vitro fertilization (IVF) and embryo transfer to isolate the effects of maternal obesity during either the gamete or gestational stages and examined how these exposures contribute to behavioral, transcriptomic, and epigenomic features relevant to ASD in first-generation offspring.

## 2. Materials and Methods

### 2.1. Study Design

**Figure 1:**
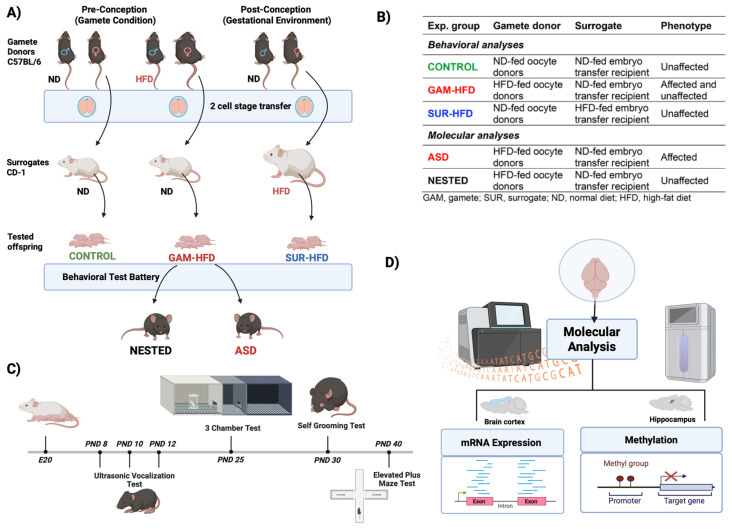
Experimental design. (**A**) Schematic representation of the experimental design. Three main groups were generated using IVF and two-cell-stage embryo transfer. Oocytes from either high fat diet (HFD)-fed or normal diet (ND)-fed female C57BL/6J mice were fertilized in vitro with sperm from ND-fed C57BL/6J males. Resulting two-cell embryos were transferred into pseudopregnant CD-1 surrogate females fed either the ND or HFD. Offspring were delivered naturally and cross-fostered at birth by ND-fed CD-1 dams to standardize postnatal maternal care. (**B**) Group stratification used for downstream analyses. Three IVF-derived experimental groups were defined by the gamete donor and surrogate environment. Behavioral phenotyping was used to classify male offspring into the ASD or unaffected subgroups. From the GAM-HFD group, male offspring were further stratified as either ASD (affected) or NESTED (unaffected) based on behavioral outcomes. (**C**) Behavioral testing schedule. A battery of ASD-relevant behavioral assays was conducted: USV on PND 8, 10, and 12; the three-chamber test for social interaction on PND 25; the self-grooming test for repetitive behavior on PND 30; and the elevated plus maze test for anxiety-like behavior on PND 40. Tests were spaced by at least 24 h to minimize stress. (**D**) Molecular analyses were performed on PND 41. Cortical tissue was used for mRNA expression and alternative splicing analysis via RNA-Seq; hippocampal tissue was used for genome-wide DNA methylation profiling using WGBS. Analyses were performed on three representative male animals per group (CONTROL, NESTED, and ASD). Created with Biorender.com (Science Suite Inc., Toronto, ON, Canada).

To separately evaluate the effects of pre-conceptional and gestational environments on offspring neurodevelopment, we used an IVF and embryo transfer design. Female oocyte donor mice (GAM *n* = 28) or surrogate embryo recipients (SUR *n* = 40) were fed either a high fat diet (HFD) or normal diet (ND), and three primary groups were established (Figure 1A,B): (1) CONTROL—oocytes and sperm from ND-fed C57BL/6 mice, transferred to ND-fed CD-1 surrogates; (2) GAM-HFD—oocytes from HFD-fed females and sperm from ND-fed males, transferred to ND-fed surrogates; and (3) SUR-HFD—oocytes and sperm from ND-fed mice, transferred to HFD-fed surrogates. The IVF-derived offspring were monitored through postnatal day (PND) 41 and subjected to a battery of behavioral assays for sociability, communication, and repetitive behaviors (Figure 1C). To avoid littermate bias, each experimental group was composed of mice that originated from 4–5 different foster litters. At a minimum, seven male and female offspring selected at random were included in each experimental group, as detailed below. The scope of this study was limited to first-generation (F1) offspring.

### 2.2. Chemicals

Equine chorionic gonadotropin (eCG; catalog HOR-272) and human chorionic gonadotropin (hCG; catalog HOR-250) were purchased from ProSpec (East Brunswick, NJ, USA). All other chemicals were obtained from Sigma-Aldrich (St. Louis, MO, USA) unless otherwise noted.

### 2.3. Animals

C57BL/6 mice (Jackson Laboratory, strain code 000664) are an inbred mouse strain with low genetic variability, which enables controlled experimental conditions and enhances reproducibility, particularly when assessing genetic or epigenetic changes; these mice were used as oocyte and sperm donors. CD-1 mice (originating from Charles River Laboratories, strain code 022) are an outbred strain known for its high reproductive success; females from this strain are good mothers and are commonly used as surrogates and foster mothers to support optimal embryo development and postnatal care. In this study, these mice were used as embryo transfer recipients and stimulus animals during behavioral testing. Mice were housed in a temperature-controlled environment (22 °C) under a 14-h light/10-h dark cycle in accordance with the University of Hawaii’s Laboratory Animal Services guidelines and protocol #14-1941-2, as approved by the Institutional Animal Care and Use Committee [16].

### 2.4. Diets

At three weeks of age, female mice were randomly assigned to receive either the HFD (D12451; 45% kcal fat) or ND (D12450J; 10% kcal fat) chow (Research Diets, NJ, USA) ad libitum for 8–10 weeks. Diets were stored at 4 °C and replaced weekly to maintain consistency. Diet effectiveness was confirmed by weight gain differences between groups (Figure S1), similar to other studies [2].

### 2.5. IVF and Embryo Transfer

To induce superovulation, oocyte donor females received 5 IU of equine chorionic gonadotropin (eCG), followed 48 h later by 5 IU of human chorionic gonadotropin (hCG). Oocytes were harvested and fertilized in vitro. Gametes were co-incubated in T6 medium, and fertilized zygotes were cultured in CZB medium until the 2-cell stage [17,18,19]. Three IVF experiments (IVF 1–3) were independently performed for both experimental groups (GAM-HFD and GAM-ND). For each IVF, oocytes from 4–5 donor females within each dietary group were pooled and fertilized using sperm from a male randomly selected from a group of available male donors. Embryos were surgically transferred into the oviducts of randomly selected pseudopregnant CD-1 females fed either the ND or HFD. Naturally derived pups were cross-fostered on the day they were born by placing them with CD-1 dams that delivered litters on the same day. Fostered litters were maintained at size of 5–6 pups per litter. For behavioral analyses, each experimental group for behavioral analyses was composed of mice that were genetically diverse and variable regarding the foster litters they were raised within. Additional details are shown in Tables S1 and S2.

### 2.6. Behavioral Analyses

#### 2.6.1. Test Battery and Scoring

IVF-derived offspring were subjected to a standardized behavioral test battery beginning on PND 8 and concluding on PND 40 (Figure 1C). The tests included the ultrasonic vocalization (USV) test on PND 8, 10, and 12; the three-chamber test on PND 25; the self-grooming test on PND 30; and the elevated plus maze (EPM) test on PND 40 [20,21,22]. Behavioral assessments were video-recorded and analyzed using Observer XT software (Noldus, Leesburg, VA, USA). Two scorers, blinded to group allocation, independently analyzed all behavioral trials. Scoring reliability was confirmed at >90% concordance; discrepancies were resolved by discussion or by using a third scorer if needed, as in our previous studies [23]. Prior to each test, mice were moved to an experimental room with recording equipment and given sufficient time (around 10 min) to acclimatize to the new environment. This procedure was consistently followed across all groups and testing time points. Additionally, animals were gently handled for 2–3 min per day over three consecutive days prior to behavioral testing to reduce stress and habituate them to human contact. All testing was conducted under standard vivarium temperature and light conditions (22 °C, 14 h light/12 h dark) using ambient light levels consistent with institutional animal care protocols. The light intensity was maintained uniformly across all experimental groups and throughout the testing period.

#### 2.6.2. Ultrasonic Vocalization Test

To assess early communication behavior, pups were isolated from the dam and placed in a bedding-lined polypropylene cage inside a sound-attenuating chamber. USV calls were recorded for 5 min using a CM16/CMPA condenser microphone placed ~10 cm above the cage. The signal was acquired using the UltrasoundGate 116H system and visualized with Recorder USGH software version (v) 4.2 (Avisoft Bioacoustics, Glienicke/Nordbahn, Germany). Files were analyzed using Avisoft SASLab Pro software (Avisoft Bioacoustics, Glienicke/Nordbahn, Germany) with a 512 FFT, Hamming window, and 75% overlap, resulting in ~488 Hz frequency resolution [24,25].

#### 2.6.3. Three-Chamber Test

Sociability was assessed using a three-chamber apparatus. On PND 25, each mouse was habituated for 10 min, followed by a 10 min test phase in which a novel CD-1 mouse was placed under one wire cup and the other cup was left empty. Entry into each chamber and the time spent were recorded and scored. Stimulus mice were sex- and age-matched and alternated across trials [26].

#### 2.6.4. Self-Grooming Test

Self-grooming behavior, a proxy for motor stereotypies, was evaluated on PND 30 by placing each mouse into a transparent Plexiglas cage (14 × 7 × 30 cm) for 10 min. Two recording devices were positioned on adjacent sides of the cage to capture both frontal and side views of the mouse’s behavior. Grooming frequency and body- part-specific duration were recorded using dual-angle cameras and later scored using ethological criteria [27,28].

#### 2.6.5. Elevated Plus Maze Test

Anxiety-like behavior was assessed on PND 40 using an elevated plus maze with two open and two closed arms. Mice were placed on the central platform and observed for 10 min. Time spent in each arm was recorded. Cleaning with 70% ethanol was performed between tests to avoid olfactory cues [29,30].

### 2.7. Molecular Analyses

#### 2.7.1. Brain Collection and DNA/RNA Extraction

On PND 41, the mice were euthanized, and the cortex (responsible for the higher-order cognitive function and implicated in ASD pathophysiology) and hippocampus (known for its sensitivity to environmental influences, including epigenetic changes), were rapidly dissected on ice and stored at −80 °C. Both the cortex and hippocampus arise from the neural tube, which is derived from the embryonic ectoderm [31,32]. Neurons in these regions exhibit similar patterns of differential DNA methylation in transcription factors and their binding motifs [33]. RNA was extracted from the cortex, and genomic DNA from the hippocampus from the indicated groups was extracted using the Qiagen AllPrep DNA/RNA/miRNA Mini Kit. Quantification was performed using Qubit Fluorometer 2.0 (Invitrogen, Carlsbad, CA, USA) with broad-range fluorescence assays.

#### 2.7.2. Transcriptomic and Methylomic Pipeline for Brain Tissue

Following standard omics study protocols [34], molecular analyses were performed using pure, high-quality RNA and DNA samples obtained from three mice per group, which served as biological replicates. As illustrated in Figure 1D, cortical RNA (100 ng per sample) was sequenced using the Illumina TruSeq stranded mRNA protocol. Reads were filtered (Q ≥ 20) and aligned to the GRCm38-mm10 genome using STAR v 2.7.10a [35]. Expression quantification was performed with RSEM v 1.3 [36]. Differential gene expression (DGE) was assessed using DESeq2 v 1.28 [37] with absolute log_2_ fold change ≥ 1 and FDR < 0.05 or 0.1. Differential transcript usage (DTU) was visualized using transcript per million (TPM) data plotted as isoform-level percentages (IsoPct), as showing the relative abundance of each isoform within a gene in a heatmap, as generated in Python v 3.11. Gene set enrichment analysis (GSEA) was performed using clusterProfiler v 4.8.3 [38], focusing on functional enrichment at the Gene Ontology/Biological Process (GO:BP) level [39] and pathways from the Kyoto Encyclopedia of Genes and Genomes (KEGG) [40] filtered at an adjusted *p* < 0.05. Gene lists were compared against the Simons Foundation Autism Research Initiative (SFARI) database (accessed March 2025) [41] and Mouse Genome Informatics (MGI) phenotype annotations [42]. Whole-genome bisulfite sequencing (WGBS) was conducted by Macrogen Inc. on 250 ng of hippocampal DNA from each sample. Bisulfite reads were aligned using Bismark v 0.24.0 [43]. Differentially methylated regions (DMRs) were identified using MethylKit v 1.22.0 [44], DSS v 2.54.0 [45], and SeqMonk v 1.48.1 (Babraham Bioinformatics). Methylation comparisons focused on the ±2 kb region around transcription start sites, promoter regions, CpG islands, and known cis-regulatory elements. Wilcoxon tests were used for statistical comparison. To assess the relationship between methylation and gene expression, methylation percentages were overlaid with mRNA expression levels using Integrative Genomic Viewer (IGV v 2.19.1, Broad Institute, Cambridge, MA, USA) with normalized bed or bedGraph files.

### 2.8. Statistical Analyses

Statistical analyses of body weight, behavior, and molecular data were performed using one-way or two-way ANOVA, followed by Tukey’s multiple comparison tests. Differences were considered statistically significant at *p* < 0.05. Analyses were performed using GraphPad Prism v 9.0 (GraphPad Software, LLC, La Jolla, CA, USA), R v 4.4.0, and Python v 3.12, as specified in the corresponding figure legends.

## 3. Results

### 3.1. Maternal Obesity Prior to Conception Induces ASD-like Behaviors in Male Offspring with Variable Penetrance

To differentiate the effects of maternal obesity before versus during pregnancy, we employed an IVF-based mouse model using C57BL/6 gamete donors and CD-1 surrogate mothers (Figure 1A,B). Female mice were fed either the HFD or the ND for 10 weeks prior to IVF and embryo transfer. Donor (GAM) and surrogate (SUR) groups showed significant weight differences after diet exposure, validating the model (Figure S1).

**Figure 2:**
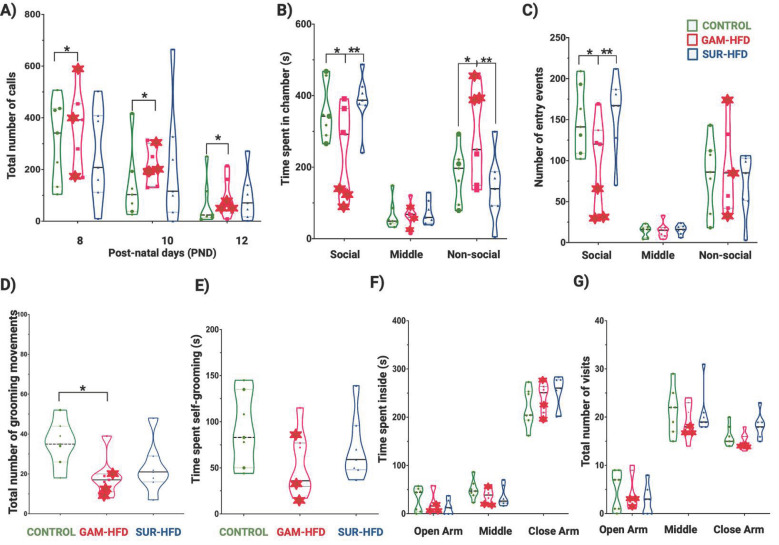
Male offspring derived from IVF with oocytes from obese females exhibit behavioral deficits associated with ASD-like phenotypes. (**A**) USV testing was performed on PND 8, 10, and 12 to assess early-life communication. Compared to CONTROL, GAM-HFD offspring emitted significantly more calls at PND 8, but significantly fewer calls by PND 10. No differences were observed in the SUR-HFD group. (**B**,**C**) In the three-chamber test for social interaction, GAM-HFD mice spent significantly less time in the social chamber and more time in the non-social chamber compared to both CONTROL and SUR-HFD groups (**B**) and exhibited significantly fewer social approach events (**C**), indicating deficits in social preference. (**D**,**E**) In the self-grooming test for repetitive behavior, GAM-HFD mice showed a significant reduction in grooming frequency (**D**) compared to CONTROL, although grooming duration (**E**) did not differ significantly across groups. The SUR-HFD group showed no differences compared to the CONTROL group. (**F**,**G**) In the elevated plus maze test, to assess anxiety-related behavior, no significant group differences were observed in the total time spent in (**F**), or the number of entries into (**G**) the open, middle, or closed arms, suggesting that anxiety-like behavior was unaffected. All tests were conducted with male offspring from the CONTROL, GAM-HFD, and SUR-HFD groups (*n* = 7 per group). Data are shown as the mean ± SD. Each dot represents an individual animal; red asterisks denote GAM-HFD individuals exhibiting the strongest ASD-like behavioral profile (see Table S2). Statistical analysis was performed using one-way or two-way ANOVA followed by Tukey’s post hoc test. * *p* < 0.05, ** *p* < 0.01.

On average, male offspring derived from obese oocyte donors (the GAM-HFD group) exhibited multiple ASD behavioral abnormalities, including altered vocalizations, social deficits, and increased self-grooming, whereas offspring from obese surrogates (SUR-HFD) did not differ significantly from the CONTROL group (Figure 2 and Table S2). In USV assays, GAM-HFD pups emitted significantly more calls at PND 8 and fewer at PND 10 compared to CONTROL pups (*p* = 0.003, Figure 2A). In the three-chamber sociability test, GAM-HFD mice spent less time interacting with the stimulus mouse and more time in the non-social chamber relative to those in both the CONTROL and SUR-HFD groups (*p* < 0.05, Figure 2B,C). Although grooming events were reduced in GAM-HFD mice (*p* = 0.032), overall grooming duration did not differ significantly between groups (Figure 2D,E). Anxiety-like behavior, assessed by the EPM test, was unaffected (Figure 2F,G), ruling out anxiety as a confounder. Female offspring across all experimental groups showed no differences in measures of vocalization or social interaction, which are primary ASD-relevant behaviors (Figure S2). Given this result, and to remain within the scope and statistical design of our study, we focused subsequent transcriptomic and epigenomic analyses exclusively on male offspring.

Due to the interindividual behavioral heterogeneity observed, GAM-HFD mice were stratified post hoc into the ASD-like phenotype (hereafter referred to as ASD) and “NESTED” subgroups based on the severity and domain breadth of phenotypic abnormalities (Figure 1A,B, Table S2). Mice in these subgroups were used in subsequent molecular analyses.

### 3.2. Transcriptional Dysregulation in ASD-like Offspring Reflects Human ASD Pathways

Given the behavioral phenotypes observed above, we next examined transcriptomic changes in cortical tissue from ASD, NESTED, and CONTROL male mice using RNA-Seq (Figure 1D). Hierarchical clustering and DGE analysis identified distinct expression profiles in ASD mice, particularly for genes associated with neurodevelopment and behavior.

**Figure 3:**
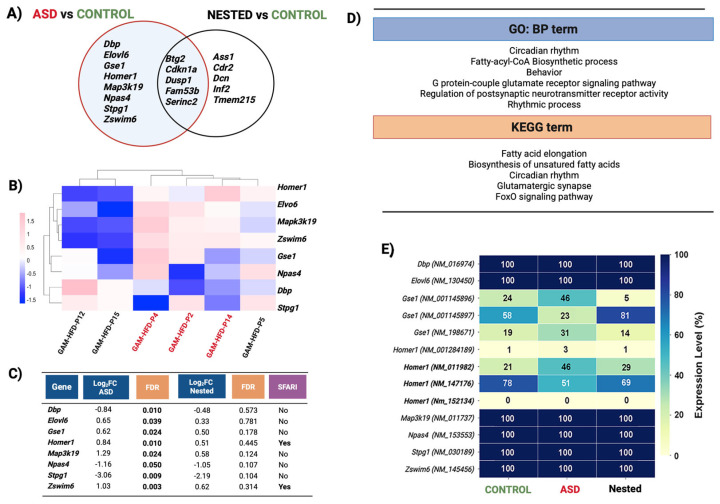
Cortical gene expression patterns distinguish behavioral phenotypes in male offspring. Transcriptomic profiling of cortical tissue was performed in three experimental subgroups (*n* = 3 per group): CONTROL, GAM-HFD ASD (ASD), and GAM-HFD Nested (NESTED). (**A**) Venn diagram showing differentially expressed genes (DEGs) between the ASD and NESTED groups compared to the CONTROL group. Eight genes that are uniquely dysregulated in the ASD group in comparison to the CONTROL group are highlighted as potential contributors to the observed ASD-like phenotype, unrelated to conditions shared with the NESTED group. (**B**) Heatmap of cortical DEGs (log_2_ fold change ≥ ±1, FDR < 0.05) across the three groups, illustrating gene-level expression patterns aligned with behavioral clustering. (**C**) Summary table of log_2_ fold change (Log_2_FC), false discovery rate (FDR), and SFARI (Simons Foundation Autism Research Initiative) database annotations for ASD-relevant genes (January 2025 release, v 3.0). (**D**) Gene set enrichment analysis (GSEA) of DEGs in the ASD vs. CONTROL group revealed enrichment in biological processes related to circadian rhythm, neurotransmission, and fatty acid biosynthesis, consistent with GO:BP and KEGG pathway annotations. (**E**) Differential transcript usage (DTU) analysis of eight key genes shows relative expression of transcript isoforms across the three groups. Expression was normalized using transcripts per million (TPM) and visualized as percentage usage per gene for each indicated group.

A relaxed statistical threshold revealed 23 genes that were exclusively altered in the ASD group (Table S3, Figure S3), while a more conservative cutoff (log_2_ fold change ≥ ±1, FDR < 0.05) identified 13 significant genes. Of these, eight genes (*Dbp*, *Elovl6*, *Gse1*, *Homer1*, *Map3k19*, *Npas4*, *Stpg1*, and *Zswim6*) were uniquely dysregulated among the ASD group and not in the NESTED group (Figure 3A–C).

Notably, of these eight genes, only *Homer1* and *Zswim6* are listed in the SFARI Gene database (retrieved: January 2025 release, v 3.0) [41]. Gene ontology and pathway analysis highlighted enriched functions related to postsynaptic receptor regulation, circadian rhythm, and fatty acid biosynthesis (Figure 3D, Table S3). NESTED mice showed mild changes in expression, suggesting that transcriptional reprogramming is associated with overt behavioral manifestation rather than exposure alone. Transcript usage analysis showed isoform-specific shifts of *Homer1* and *Gse1* in ASD mice, reinforcing the functional significance of transcript-level regulation (Figure 3E).

### 3.3. Molecular Interaction Networks Reveal ASD-Specific Signaling Alterations

**Figure 4:**
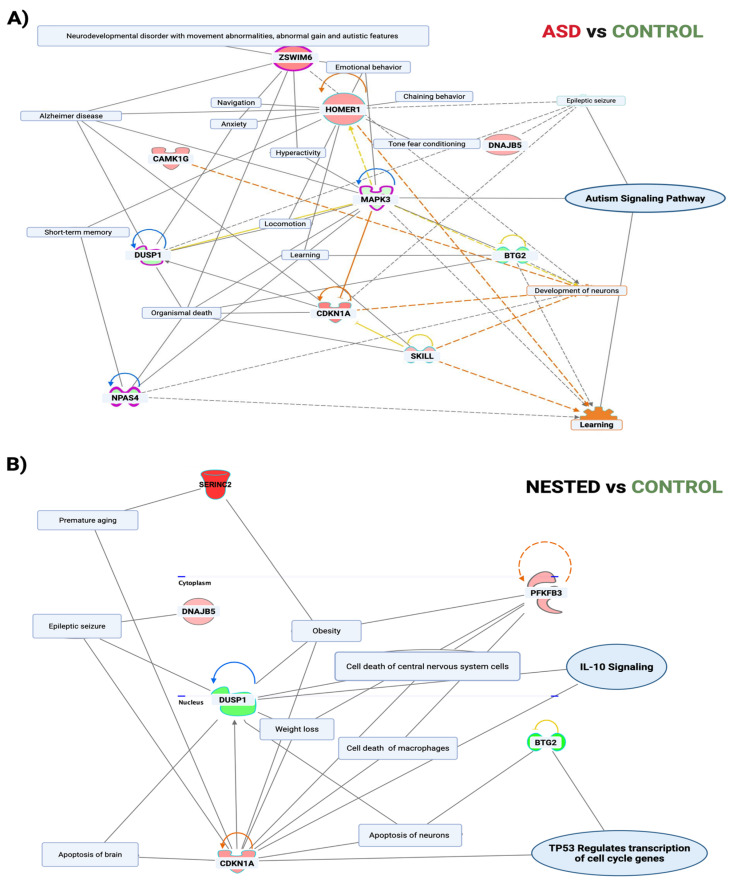
Co-expression networks reveal distinct molecular programs in the cortex of male offspring with ASD-like vs. unaffected phenotypes. (**A**) Weighted gene co-expression network analysis (WGCNA) was applied to cortical transcriptomes from male offspring to identify molecular modules associated with ASD-like traits. In ASD-classified GAM-HFD offspring, a highly connected co-expression module emerged and centered on *Homer1*, a synaptic scaffolding gene previously implicated in autism. This module was enriched for multiple ASD candidate genes curated in the SFARI Gene database (v 3.0), including *Zswim6*, *Npas4*, *Map3k19*, and *Cdkn1a*. Network topology reflects gene–gene correlations (edges; *p* ≥ 0.05), with node size proportional to intramodular connectivity (kME) and font size reflecting relative hub status. (**B**) In contrast, male offspring from the same IVF and prenatal conditions that did not exhibit ASD-like behaviors (“NESTED” subgroup) displayed a distinct co-expression module lacking ASD-related genes. Instead, this network was enriched for pathways involved in inflammation (IL-10 signaling), cellular stress, apoptosis (*Cdkn1a*, *Serinc2*), metabolic adaptation (*Pfkb3*, *Dusp1*), and aging. This divergent expression profile may reflect compensatory mechanisms that mitigate neurodevelopmental risk in the absence of overt behavioral abnormalities. This analysis was performed using Ingenuity Pathway Analysis (QIAGEN Inc., Venlo, The Netherlands) with the Enrichment module, filtered for brain-relevant phenotypes and functions. Only pathways with a Benjamini–Hochberg adjusted *p*-value < 0.05 and absolute Z-score > 2 were retained. Network structure and biological terms are annotated to highlight phenotype-specific regulatory programs.

Pathway analysis of differentially expressed genes in the ASD group identified molecular networks linked to nervous system disorders, synaptic plasticity, and the canonical autism signaling pathway, involving key nodes such as *Mapk3* and *Homer1* (Figure 4A). In contrast, networks in the NESTED group were enriched for pathways related to metabolic regulation, neural apoptosis, and premature aging (Figure 4B), consistent with epileptic seizure comorbidities in humans.

### 3.4. Aberrant Patterns of DNA Methylation Within the Homer1 Promoter Region Are Associated with the ASD Phenotype

Neuronal cells from both the cortex and hippocampus exhibit dynamic DNA methylation changes at transcription factor binding sites. The hippocampus—a neurogenic region highly sensitive to environmental influences—is especially susceptible to epigenetic modulation. Multiple studies have demonstrated that environmental exposures during critical periods of development can induce region-specific changes in DNA methylation at transcription factor binding sites and their regulatory elements, potentially altering gene expression programs in both cortical and hippocampal circuits [33]. We previously observed aberrant DNA methylation patterning in an adult neurogenic region of ASD brain resembling earlier fetal-like states [14,15]. Thus, to capture potential changes to DNA methylation states that may have occurred early in neurodevelopment and persisted in adolescent male offspring (PND 41), we focused on profiling hippocampal tissue.

**Figure 5:**
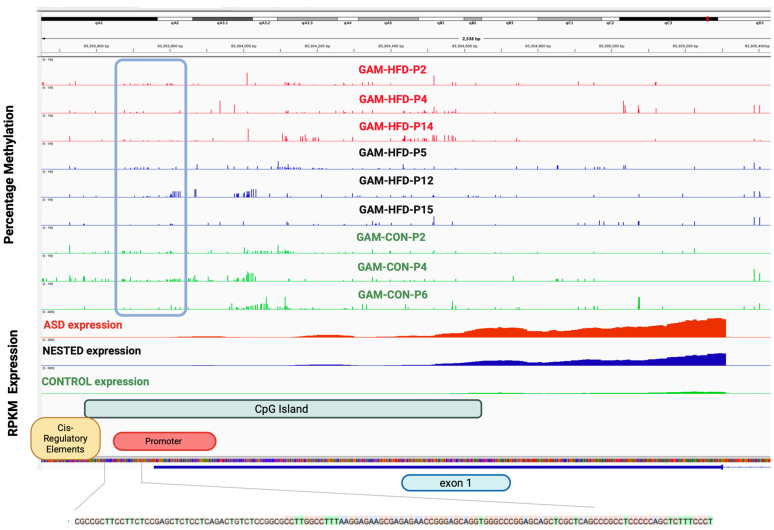
Differential DNA methylation at an alternative promoter of *Homer1* correlates with isoform-specific expression. Significant group-specific differences in DNA methylation were observed within the promoter and exon 1 region of *Homer1*, a locus associated with synaptic scaffolding and plasticity. Two distinct promoter regions were identified: the canonical promoter (5′) and an alternative downstream promoter overlapping a CpG island and a *cis*-regulatory element (CRE). While no significant differences were found in methylation at the canonical promoter across groups (Kruskal–Wallis test, χ^2^ = 0.96, *p* = 0.618; mean methylation: CONTROL = 6.17%, ASD = 4.83%, NESTED = 4.0%), the alternative promoter showed marked hypomethylation in the ASD group. Specifically, the alternative promoter was hypermethylated in the CONTROL group and one NESTED sample, but completely unmethylated in ASD mice (Fisher’s exact test, *p* = 0.01). This regional hypomethylation corresponded with increased expression of *Homer1a*, the short, activity-inducible isoform, in ASD offspring (mean RPKM: ASD = 2800 ± 280 vs. CONTROL = 200 ± 50; unpaired two-tailed *t*-test, *p* = 0.001). While the WGBS and RNA-Seq data were derived from distinct brain regions (hippocampus and cortex, respectively), the observed pattern suggests a potentially conserved epigenetic regulatory mechanism. However, we note that this correlation is indirect given the different tissue types evaluated. Equally scaled tracks show the percentage of DNA methylation (top) and normalized RNA expression (RPKM, bottom) across individual samples for each group (GAM-HFD, NESTED, and CONTROL). The genome architecture includes annotated *cis*-regulatory elements, the CpG island, and exon 1. The base-level nucleotide positions of the analyzed CRE-containing CpGs are displayed below.

Whole-genome bisulfite sequencing (WGBS) revealed no significant global methylation differences across the GAM-HFD (ASD and NESTED) and CONTROL groups (Figure S4). However, we observed region-specific differences in DNA methylation, particularly at genes previously identified as transcriptionally dysregulated. Notably, the *Homer1* locus exhibited distinct methylation patterns at its promoter regions. Although the canonical promoter remained largely unchanged across groups, the alternative promoter displayed striking group-specific differences, being unmethylated in the hippocampus of ASD mice, hypermethylated in CONTROL mice, and partially methylated in NESTED mice (Figure 5). This differential methylation pattern was associated with elevated expression of the short, activity-inducible isoform *Homer1a*, which is known to disrupt postsynaptic scaffolding and modulate synaptic plasticity. The increased expression of *Homer1a* in ASD-classified mice suggests that pre-conceptional obesity may alter isoform expression through epigenetic derepression of an alternative promoter. Notably, our previous work has demonstrated that DNA methylation plays a critical role in regulating alternative promoter usage of neurodevelopmental genes, including *SHANK3*, a gene functionally related to *Homer1* and independently implicated in ASD [46].

### 3.5. Visual Summary: Maternal Obesity Alters Neural Gene Regulation and Behavior

Collectively, our results show that pre-conception maternal obesity alone is sufficient to drive ASD behaviors and brain transcriptomic changes in male offspring. Behavioral severity was correlated with altered expression and isoform usage of *Homer1*, which was epigenetically regulated at an alternative promoter. These findings identify a critical window where maternal environment programs long-term neurodevelopmental outcomes via transcriptional and epigenetic reprogramming (graphical abstract).

## 4. Discussion

### 4.1. Pre-Conception Obesity Reprograms Neurodevelopment and Induces ASD Traits

ASD diagnosis in humans relies on behavioral assessments and developmental history, often delaying intervention due to the lack of molecular biomarkers. Increasing evidence suggests that early-life epigenetic dysregulation may precede symptom onset and contribute to ASD etiology [1,2,15,41,47]. Our findings support this hypothesis by showing that pre-conception maternal obesity alone can induce behavioral and molecular phenotypes in F1 mice that closely resemble core features of ASD [1,2,11,48].

Using an IVF-based approach, we disentangled the influence of maternal obesity before conception from effects during gestation, with a focus on early developmental events in first-generation offspring. Female F1 offspring did not exhibit any significant ASD phenotype in this study, which is consistent with the lower prevalence of ASD among females in the human population [1,2,3]. In contrast, behavioral testing revealed that male offspring derived from obese oocyte donors—but not those gestated in obese surrogates—exhibited ASD deficits in communication, sociability, and repetitive behaviors [6,7]. This supports prior epidemiological studies linking maternal metabolic health to ASD risk and highlights the pre-conception window as a critical period for programming neurodevelopmental outcomes [6,7,8,9,10].

### 4.2. Behavioral Domains Are Selectively Altered by Pre-Conceptional Programming

Behavioral assays revealed that communication, sociability, and repetitive behavior domains were selectively disrupted in a subset of male offspring derived from oocytes of obese females (the GAM-HFD group), supporting the notion of pre-conceptional metabolic programming. USV analysis demonstrated altered calling patterns in GAM-HFD pups compared to that of CONTROL pups, reminiscent of the delayed vocal development frequently observed in children with ASD [49]. Similarly, performance in the three-chamber test for sociability revealed significant reductions in social engagement, aligning with the social withdrawal and avoidance phenotypes reported in both ASD mouse models and human clinical populations [27,50,51]. Increased frequency of grooming movements—an established surrogate for repetitive, stereotypic behavior—further reinforced the presence of ASD-like phenotypes [23,26]. These behavioral alterations occurred in the absence of differences in anxiety-like behavior as assessed by the elevated plus maze test, thereby excluding general stress or anxiety as a confounding factor [52].

Altogether, these findings recapitulate the triad of core behavioral domains used to define ASD: impaired communication, reduced social interaction, and repetitive behaviors [53]. Interestingly, offspring from the SUR-HFD group (gestational obesity only) did not exhibit any consistent behavioral abnormalities, indicating that maternal obesity during gestation, in isolation, is insufficient to induce ASD-like traits. The coexistence of behaviorally unaffected “NESTED” mice within the GAM-HFD group—despite identical pre-conceptional and gestational exposures—highlights the phenotypic heterogeneity of ASD and supports the contribution of individual susceptibility, consistent with observations in humans [1,2,3].

### 4.3. Cortical Gene Expression Changes Mirror Known ASD Risk Pathways

Transcriptomic analysis of cortex tissue revealed distinct gene expression alterations uniquely associated with ASD-affected offspring. Eight differentially expressed genes (DEGs) were identified exclusively in this group, including *Homer1* and *Zswim6*, both of which are listed as high-confidence autism risk genes in the SFARI Gene database [41]. Notably, *Homer1* encodes a synaptic scaffolding protein that regulates excitatory neurotransmission and synaptic plasticity, while *Zswim6* mutations have been linked to neurodevelopmental delay and ASD-like features. Other significantly dysregulated genes, such as *Dbp*, *Map3k19*, and *Npas4*, are involved in circadian rhythm regulation, neuronal excitability, and activity-dependent synaptic modulation [54,55,56,57]. These molecular pathways overlap substantially with established ASD-related processes, including disruptions in homeostatic excitation/inhibition balance and the temporal coordination of gene expression that is critical for neurodevelopment.

Interestingly, the same genes exhibited only modest or statistically nonsignificant changes in the NESTED group, suggesting that transcriptional dysregulation may reflect the behavioral severity observed in the ASD subgroup. This supports a dosage-sensitive or threshold-dependent model of neurodevelopmental vulnerability, in which cumulative changes in gene expression—rather than the presence of a single alteration—drive ASD phenotypes [13,58]. Moreover, differences in transcript isoform usage observed across groups further imply that alternative splicing may contribute to phenotypic diversity by subtly modulating protein function and neuronal circuit assembly.

### 4.4. Epigenetic Regulation of Homer1 Isoforms Links Molecular and Behavioral Phenotypes

One of the most striking molecular findings of this study was the isoform-specific epigenetic regulation of the *Homer1* gene, particularly at its alternative promoter region. In ASD mice, we observed a complete loss of DNA methylation in this region, which was strongly associated with elevated expression of the short, activity-inducible isoform *Homer1a*. This correlation—while derived from separate tissue samples—is consistent with prior reports implicating *Homer1a* in the disruption of synaptic scaffolding and the attenuation of mGluR-mediated signaling, both of which are central to the altered synaptic plasticity and connectivity observed in neurodevelopmental disorders [59,60,61,62,63,64].

In contrast, no significant DNA methylation differences were detected at the canonical *Homer1* promoter, underscoring the specificity of epigenetic changes to the alternative regulatory region. This selective hypomethylation pattern was unique to ASD mice and was not present in either the NESTED or CONTROL groups, suggesting a mechanistic link between epigenetic dysregulation and severity of ASD-like behavioral phenotypes. Given its established role in activity-dependent synaptic remodeling, *Homer1a* emerges as a compelling candidate for biomarker development and therapeutic targeting in ASD.

The observed promoter-specific DNA methylation changes align with previous studies in models of major depressive disorder and other stress-responsive conditions, suggesting that dysregulation of *Homer1a* may reflect a broader vulnerability mechanism in neuropsychiatric and neurodevelopmental disorders [61]. While these findings provide compelling preliminary evidence, further validation is required, including allele-specific methylation, isoform-specific transcriptional quantification, and causal manipulation, to conclusively establish *Homer1a* as an epigenetically regulated effector of ASD phenotypes.

### 4.5. Implications, Limitations, and Future Directions

Our study demonstrates that maternal obesity prior to conception can lead to lasting epigenetic alterations in the oocyte that subsequently influence transcriptional regulation in the developing brain of first-generation offspring. These changes are associated with ASD-like behaviors in male offspring, and our findings specifically highlight isoform-specific dysregulation of the synaptic gene *Homer1*—notably the increased expression of the activity-inducible *Homer1a* isoform—as a potential molecular signature of susceptibility. This observation underscores the importance of precise transcriptional control in neurodevelopment and suggests that epigenetically mediated isoform shifts may contribute to the emergence of ASD-relevant phenotypes.

These results carry several key implications. First, they point to maternal metabolic status before pregnancy as a critical and potentially modifiable environmental risk factor for neurodevelopmental disorders. This finding supports the growing literature on the developmental origins of health and disease and underscores the value of pre-conception care as a strategy for reducing long-term neurodevelopmental risk in offspring. Second, our data suggest that specific molecular signatures, such as promoter DNA methylation and isoform expression patterns in genes such as *Homer1*, may serve as early biomarkers of atypical neurodevelopment or potential targets for intervention.

However, several limitations of the current study should be acknowledged. While assisted reproductive technology (ART) procedures such as superovulation and IVF can induce epigenetic changes, all offspring in this study were generated using identical ART protocols, ensuring equivalent exposure across groups. Thus, the observed transcriptional and epigenetic differences are attributed to biological variation related to maternal obesity rather than procedural artifacts. The transcriptomic and DNA methylation analyses were performed on bulk tissue from the cortex and hippocampus, precluding cell-type-specific resolution. Molecular profiling was conducted in a relatively small sample set, which, while appropriate for mechanistic insight, may limit statistical power. Furthermore, behavioral and molecular analyses were restricted to male offspring due to the absence of observable ASD-like traits in females; future investigations should assess sex-specific vulnerability and resilience more comprehensively. In addition, although we observed strong correlations between regional promoter DNA methylation and transcript expression, causality remains to be confirmed through functional manipulation studies. In addition, our study focuses on a relatively short postnatal follow-up period, ending at PND 41. It remains unknown whether the observed behavioral and molecular phenotypes persist into adulthood or resolve over time. Finally, while it was not possible to match specific oocyte donors to offspring due to the pooling strategy in IVF, our design allowed us to isolate gametic versus gestational contributions.

Future studies will consider parallel profiling of donor females to determine whether gene expression changes in ASD-classified offspring are inherited or newly programmed during early development, as well as examine late adolescent and adult time points that will be critical to determine the stability and developmental trajectory of ASD-related traits induced by pre-conceptional maternal obesity. Such studies should aim to map the cell-type-specific methylation and gene expression landscapes in the developing brain and assess the potential reversibility of transcriptomic imbalances. Interventions that target epigenetic modulators—through dietary, environmental, or pharmacological strategies—may offer new opportunities for preventing or attenuating neurodevelopmental risk. Expanding sample sizes and tracking long-term neurobehavioral outcomes, transgenerationally, will also be essential to improving generalizability and translating these findings to clinical relevance. Indeed, given the emerging role of epigenetic modification in transgenerational inheritance, future studies should evaluate whether the effects of maternal obesity extend to the F2 generation through persistent germline or somatic epimutations, which may further elevate ASD risk in descendants. By integrating behavioral, immunological, and biochemical parameters, as well as transcriptomics and epigenetic data within a preclinical model of maternal metabolic risk, this study provides a valuable framework for exploring modifiable pathways in ASD susceptibility and early-life brain programming.

## Data Availability

All data used for this project will be available upon reasonable request to the corresponding author. Molecular data generated in this study are available under the NCBI BioProject accession number PRJNA1299706.

## Supplementary Materials

The following supporting information can be downloaded at: https://www.mdpi.com/article/10.3390/cells14151201/s1. Figure S1: Diet-based induction of maternal obesity in donor and surrogate mice; Figure S2: Female offspring from all embryo transfer groups did not exhibit ASD-like behavioral phenotypes; Figure S3: Hierarchical clustering, differential gene expression, and gene set enrichment analyses of cortical transcriptomes from male offspring; Figure S4: Global DNA methylation landscape across hippocampal genomes of male offspring; Table S1: In vitro fertilization (IVF) and embryo transfer summary across experimental groups; Table S2: List of male offspring derived by IVF, embryo transfer and cross-fostering, and subjected to behavioral and molecular analyses; Table S3: Functional enrichment analysis of the candidate genes associated with ASD phenotype.

## Abbreviations

The following abbreviations are used in this manuscript:

ASD	Autism spectrum disorder
CP	Canonical Pathway
DGE	Differential gene expression
DOHaD	Developmental Origins of Health and Disease
DMR	Differentially methylated regions
DTU	Differential transcript usage
EPM	Elevate plus maze
FC	Fold change
GAM	Gamete Donor
GO:BP	Gene Ontology: Biological Process
GSEA	Gene Set Enrichment Analysis
HFD	High fat diet
IVF	In vitro fertilization
KEGG	Kyoto Encyclopedia of Genes and Genomes
kME	Module Eigengene-based Connectivity
MSigDB	Molecular Signatures database
ND	Normal diet
PND	Postnatal day
MGI	Mouse Genome Informatics
RPKM	Reads per kilobase of transcript, per Million mapped reads
SFARI	Simons Foundation Autism Research Initiative
SUR	Surrogate
TPM	Transcript per Millon
USV	Ultrasonic Vocalization
WGCNA	Weighted gene co-expression network analysis
WGBS	Whole-genome bisulfite sequencing

## References

[1] Nordahl C.W., Andrews D.S., Dwyer P., Waizbard-Bartov E., Restrepo B., Lee J.K., Heath B., Saron C., Rivera S.M., Solomon M. (2021). The Autism Phenome Project: Toward Identifying Clinically Meaningful Subgroups of Autism. Front. Neurosci..

[2] Maenner M.J., Warren Z., Williams A.R., Amoakohene E., Bakian A.V., Bilder D.A., Durkin M.S., Fitzgerald R.T., Furnier S.M., Hughes M.M. (2023). Prevalence and Characteristics of Autism Spectrum Disorder Among Children Aged 8 Years—Autism and Developmental Disabilities Monitoring Network, 11 Sites, United States, 2020. MMWR Surveill. Summ..

[3] Loomes R., Hull L., Mandy W.P.L. (2017). What Is the Male-to-Female Ratio in Autism Spectrum Disorder? A Systematic Review and Meta-Analysis. J. Am. Acad. Child. Adolesc. Psychiatry.

[4] Zeidan J., Fombonne E., Scorah J., Ibrahim A., Durkin M.S., Saxena S., Yusuf A., Shih A., Elsabbagh M. (2022). Global prevalence of autism: A systematic review update. Autism Res..

[5] Strathearn L., Momany A., Kovacs E.H., Guiler W., Ladd-Acosta C. (2023). The intersection of genome, epigenome and social experience in autism spectrum disorder: Exploring modifiable pathways for intervention. Neurobiol. Learn. Mem..

[6] Agarwal P., Morriseau T.S., Kereliuk S.M., Doucette C.A., Wicklow B.A., Dolinsky V.W. (2018). Maternal obesity, diabetes during pregnancy and epigenetic mechanisms that influence the developmental origins of cardiometabolic disease in the offspring. Crit. Rev. Clin. Lab. Sci..

[7] Balachandar V., Mahalaxmi I., Neethu R., Arul N., Abhilash V.G. (2022). New insights into epigenetics as an influencer: An associative study between maternal prenatal factors in Autism Spectrum Disorder (ASD). Neurol. Perspect..

[8] Banik A., Kandilya D., Ramya S., Stünkel W., Chong Y.S., Dheen S.T. (2017). Maternal Factors that Induce Epigenetic Changes Contribute to Neurological Disorders in Offspring. Genes.

[9] Bilder D.A., Bakian A.V., Viskochil J., Clark E.A., Botts E.L., Smith K.R., Pimentel R., McMahon W.M., Coon H. (2013). Maternal prenatal weight gain and autism spectrum disorders. Pediatrics.

[10] Chao S., Lu J., Li L.J., Guo H.Y., Xu K., Wang N., Zhao S.X., Jin X.W., Wang S.G., Yin S. (2024). Maternal obesity may disrupt offspring metabolism by inducing oocyte genome hyper-methylation via increased DNMTs. eLife.

[11] Lei X.Y., Li Y.J., Ou J.J., Li Y.M. (2019). Association between parental body mass index and autism spectrum disorder: A systematic review and meta-analysis. Eur. Child. Adolesc. Psychiatry.

[12] Liu X., Li X., Xia B., Jin X., Zou Q., Zeng Z., Zhao W., Yan S., Li L., Yuan S. (2021). High-fiber diet mitigates maternal obesity-induced cognitive and social dysfunction in the offspring via gut-brain axis. Cell Metab..

[13] Sinclair K.D., Lea R.G., Rees W.D., Young L.E. (2007). The developmental origins of health and disease: Current theories and epigenetic mechanisms. Soc. Reprod. Fertil. Suppl..

[14] Corley M.J., Vargas-Maya N., Pang A.P.S., Lum-Jones A., Li D., Khadka V., Sultana R., Blanchard D.C., Maunakea A.K. (2019). Epigenetic Delay in the Neurodevelopmental Trajectory of DNA Methylation States in Autism Spectrum Disorders. Front. Genet..

[15] Takahashi E., Allan N., Peres R., Ortug A., van der Kouwe A.J.W., Valli B., Ethier E., Levman J., Baumer N., Tsujimura K. (2022). Integration of structural MRI and epigenetic analyses hint at linked cellular defects of the subventricular zone and insular cortex in autism: Findings from a case study. Front. Neurosci..

[16] Prepared by the Animal Facilities Standards Committee of the Animal Care Panel (2021). Guide for Laboratory Animal Facilities and Care. ILAR J..

[17] Chatot C.L., Ziomek C.A., Bavister B.D., Lewis J.L., Torres I. (1989). An improved culture medium supports development of random-bred 1-cell mouse embryos in vitro. J. Reprod. Fertil..

[18] Riel J.M., Yamauchi Y., Ruthig V.A., Malinta Q.U., Blanco M., Moretti C., Cocquet J., Ward M.A. (2019). Rescue of Sly Expression Is Not Sufficient to Rescue Spermiogenic Phenotype of Mice with Deletions of Y Chromosome Long Arm. Genes.

[19] Quinn P., Barros C., Whittingham D.G. (1982). Preservation of hamster oocytes to assay the fertilizing capacity of human spermatozoa. J. Reprod. Fertil..

[20] Silverman J.L., Thurm A., Ethridge S.B., Soller M.M., Petkova S.P., Abel T., Bauman M.D., Brodkin E.S., Harony-Nicolas H., Wöhr M. (2022). Reconsidering animal models used to study autism spectrum disorder: Current state and optimizing future. Genes Brain Behav..

[21] Peca J., Feliciano C., Ting J.T., Wang W., Wells M.F., Venkatraman T.N., Lascola C.D., Fu Z., Feng G. (2011). Shank3 mutant mice display autistic-like behaviours and striatal dysfunction. Nature.

[22] Arakawa H., Blanchard D.C., Arakawa K., Dunlap C., Blanchard R.J. (2008). Scent marking behavior as an odorant communication in mice. Neurosci. Biobehav. Rev..

[23] Balaan C., Corley M.J., Eulalio T., Leite-Ahyo K., Pang A.P.S., Fang R., Khadka V.S., Maunakea A.K., Ward M.A. (2019). Juvenile Shank3b deficient mice present with behavioral phenotype relevant to autism spectrum disorder. Behav. Brain Res..

[24] Moy S.S., Nadler J.J., Perez A., Barbaro R.P., Johns J.M., Magnuson T.R., Piven J., Crawley J.N. (2004). Sociability and preference for social novelty in five inbred strains: An approach to assess autistic-like behavior in mice. Genes. Brain Behav..

[25] Moy S.S., Nadler J.J., Young N.B., Nonneman R.J., Segall S.K., Andrade G.M., Crawley J.N., Magnuson T.R. (2008). Social approach and repetitive behavior in eleven inbred mouse strains. Behav. Brain Res..

[26] Pearson B.L., Pobbe R.L., Defensor E.B., Oasay L., Bolivar V.J., Blanchard D.C., Blanchard R.J. (2011). Motor and cognitive stereotypies in the BTBR T+tf/J mouse model of autism. Genes. Brain Behav..

[27] Defensor E.B., Pearson B.L., Pobbe R.L., Bolivar V.J., Blanchard D.C., Blanchard R.J. (2011). A novel social proximity test suggests patterns of social avoidance and gaze aversion-like behavior in BTBR T+ tf/J mice. Behav. Brain Res..

[28] Handley S.L., Mithani S. (1984). Effects of alpha-adrenoceptor agonists and antagonists in a maze-exploration model of ‘fear’-motivated behaviour. Naunyn Schmiedebergs Arch. Pharmacol..

[29] Komada M., Takao K., Miyakawa T. (2008). Elevated plus maze for mice. J. Vis. Exp..

[30] Pobbe R.L., Defensor E.B., Pearson B.L., Bolivar V.J., Blanchard D.C., Blanchard R.J. (2011). General and social anxiety in the BTBR T+ tf/J mouse strain. Behav. Brain Res..

[31] Chen V.S., Morrison J.P., Southwell M.F., Foley J.F., Bolon B., Elmore S.A. (2017). Histology Atlas of the Developing Prenatal and Postnatal Mouse Central Nervous System, with Emphasis on Prenatal Days E7.5 to E18.5. Toxicol. Pathol..

[32] Rajan A., Fame R.M. (2024). Brain development and bioenergetic changes. Neurobiol. Dis..

[33] Liu H., Zhou J., Tian W., Luo C., Bartlett A., Aldridge A., Lucero J., Osteen J.K., Nery J.R., Chen H. (2021). DNA methylation atlas of the mouse brain at single-cell resolution. Nature.

[34] van Kampen A.H.C., Mahamune U., Jongejan A., van Schaik B.D.C., Balashova D., Lashgari D., Pras-Raves M., Wever E.J.M., Dane A.D., García-Valiente R. (2024). ENCORE: A practical implementation to improve reproducibility and transparency of computational research. Nat. Commun..

[35] Dobin A., Gingeras T.R. (2015). Mapping RNA-seq Reads with STAR. Curr. Protoc. Bioinform..

[36] Li B., Dewey C.N. (2011). RSEM: Accurate transcript quantification from RNA-Seq data with or without a reference genome. BMC Bioinform..

[37] Love M.I., Huber W., Anders S. (2014). Moderated estimation of fold change and dispersion for RNA-seq data with DESeq2. Genome Biol..

[38] Yu G., Wang L.G., Han Y., He Q.Y. (2012). clusterProfiler: An R package for comparing biological themes among gene clusters. Omics.

[39] Harris M.A., Clark J., Ireland A., Lomax J., Ashburner M., Foulger R., Eilbeck K., Lewis S., Marshall B., Mungall C. (2004). The Gene Ontology (GO) database and informatics resource. Nucleic Acids Res..

[40] Kanehisa M., Goto S. (2000). KEGG: Kyoto encyclopedia of genes and genomes. Nucleic Acids Res..

[41] Consortium T.S. (2018). SPARK: A US Cohort of 50,000 Families to Accelerate Autism Research. Neuron.

[42] Blake J.A., Baldarelli R., Kadin J.A., Richardson J.E., Smith C.L., Bult C.J., Mouse Genome Database G. (2021). Mouse Genome Database (MGD): Knowledgebase for mouse-human comparative biology. Nucleic Acids Res..

[43] Krueger F., Andrews S.R. (2011). Bismark: A flexible aligner and methylation caller for Bisulfite-Seq applications. Bioinformatics.

[44] Akalin A., Kormaksson M., Li S., Garrett-Bakelman F.E., Figueroa M.E., Melnick A., Mason C.E. (2012). methylKit: A comprehensive R package for the analysis of genome-wide DNA methylation profiles. Genome Biol..

[45] Feng H., Wu H. (2019). Differential methylation analysis for bisulfite sequencing using DSS. Quant. Biol..

[46] Maunakea A.K., Nagarajan R.P., Bilenky M., Ballinger T.J., D’Souza C., Fouse S.D., Johnson B.E., Hong C., Nielsen C., Zhao Y. (2010). Conserved role of intragenic DNA methylation in regulating alternative promoters. Nature.

[47] Yu H.Y., Zhou Y.Y., Pan L.Y., Zhang X., Jiang H.Y. (2022). Early Life Antibiotic Exposure and the Subsequent Risk of Autism Spectrum Disorder and Attention Deficit Hyperactivity Disorder: A Systematic Review and Meta-Analysis. J. Autism Dev. Disord..

[48] Takahashi T., Okabe S., Broin P.O., Nishi A., Ye K., Beckert M.V., Izumi T., Machida A., Kang G., Abe S. (2016). Structure and function of neonatal social communication in a genetic mouse model of autism. Mol. Psychiatry.

[49] Gawlińska K., Gawliński D., Borczyk M., Korostyński M., Przegaliński E., Filip M. (2021). A Maternal High-Fat Diet during Early Development Provokes Molecular Changes Related to Autism Spectrum Disorder in the Rat Offspring Brain. Nutrients.

[50] Bertelsen N., Landi I., Bethlehem R.A.I., Seidlitz J., Busuoli E.M., Mandelli V., Satta E., Trakoshis S., Auyeung B., Kundu P. (2021). Imbalanced social-communicative and restricted repetitive behavior subtypes of autism spectrum disorder exhibit different neural circuitry. Commun. Biol..

[51] Rein B., Yan Z., Wang Z.J. (2020). Diminished social interaction incentive contributes to social deficits in mouse models of autism spectrum disorder. Genes. Brain Behav..

[52] Turi M., Burr D.C., Igliozzi R., Aagten-Murphy D., Muratori F., Pellicano E. (2015). Children with autism spectrum disorder show reduced adaptation to number. Proc. Natl. Acad. Sci. USA.

[53] Das I., Estevez M.A., Sarkar A.A., Banerjee-Basu S. (2019). A multifaceted approach for analyzing complex phenotypic data in rodent models of autism. Mol. Autism.

[54] Liu H., Huang X., Xu J., Mao H., Li Y., Ren K., Ma G., Xue Q., Tao H., Wu S. (2021). Dissection of the relationship between anxiety and stereotyped self-grooming using the Shank3B mutant autistic model, acute stress model and chronic pain model. Neurobiol. Stress..

[55] Lopez-Molina L., Conquet F., Dubois-Dauphin M., Schibler U. (1997). The DBP gene is expressed according to a circadian rhythm in the suprachiasmatic nucleus and influences circadian behavior. EMBO J..

[56] Lin Y., Bloodgood B.L., Hauser J.L., Lapan A.D., Koon A.C., Kim T.K., Hu L.S., Malik A.N., Greenberg M.E. (2008). Activity-dependent regulation of inhibitory synapse development by Npas4. Nature.

[57] Rosina E., Battan B., Siracusano M., Di Criscio L., Hollis F., Pacini L., Curatolo P., Bagni C. (2019). Disruption of mTOR and MAPK pathways correlates with severity in idiopathic autism. Transl. Psychiatry.

[58] Javed S., Selliah T., Lee Y.-J., Huang W.-H. (2020). Dosage-sensitive genes in autism spectrum disorders: From neurobiology to therapy. Neurosci. Biobehav. Rev..

[59] Yoon S., Piguel N.H., Khalatyan N., Dionisio L.E., Savas J.N., Penzes P. (2021). *Homer1* promotes dendritic spine growth through ankyrin-G and its loss reshapes the synaptic proteome. Mol. Psychiatry.

[60] Banerjee A., Luong J.A., Ho A., Saib A.O., Ploski J.E. (2016). Overexpression of *Homer1a* in the basal and lateral amygdala impairs fear conditioning and induces an autism-like social impairment. Mol. Autism.

[61] Sun L., Verkaik-Schakel R.N., Biber K., Plosch T., Serchov T. (2021). Antidepressant treatment is associated with epigenetic alterations of *Homer1* promoter in a mouse model of chronic depression. J. Affect. Disord..

[62] Inoue N., Nakao H., Migishima R., Hino T., Matsui M., Hayashi F., Nakao K., Manabe T., Aiba A., Inokuchi K. (2009). Requirement of the immediate early gene vesl-1S/homer-1a for fear memory formation. Mol. Brain.

[63] Szumlinski K.K., Kalivas P.W., Worley P.F. (2006). Homer proteins: Implications for neuropsychiatric disorders. Curr. Opin. Neurobiol..

[64] Szumlinski K.K., Abernathy K.E., Oleson E.B., Klugmann M., Lominac K.D., He D.Y., Ron D., During M., Kalivas P.W. (2006). Homer isoforms differentially regulate cocaine-induced neuroplasticity. Neuropsychopharmacology.

