# Supplementary material for: Pre-Conception Maternal Obesity Confers Autism Spectrum Disorder-like Behaviors in Mice Offspring Through Neuroepigenetic Dysregulation
Source: Cells. 2025 Aug 5;14(15):1201. doi: 10.3390/cells14151201 (PMC12345829; doi:10.3390/cells14151201)
Supplement: Supplementary file 1 [file cells-14-01201-s001.zip › cells-3739855-supplementary.pdf]

Supplementary Materials

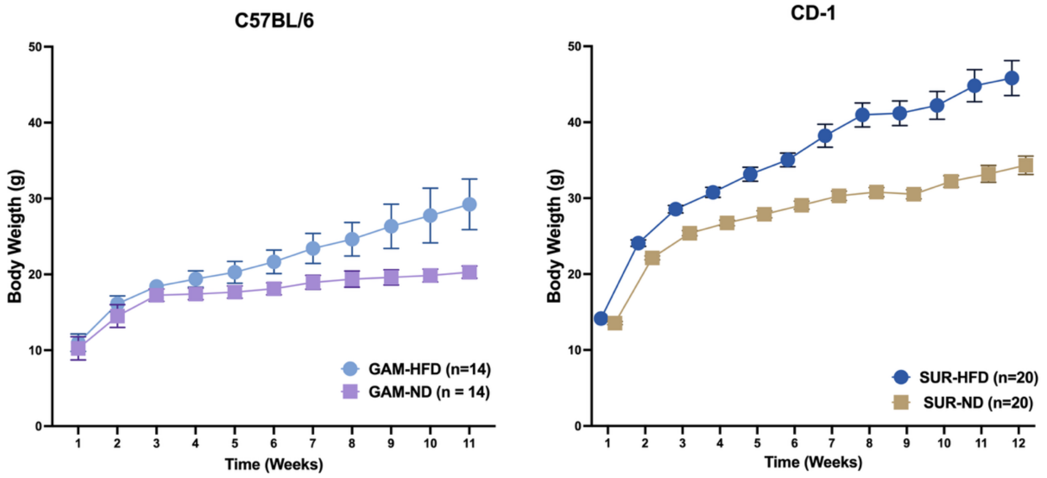

Figure S1. Diet-based induction of maternal obesity in donor and surrogate mice. Pre-conception high fat diet-induced obesity in oocyte donor and surrogate recipient mice. **(A)** Female C57BL/6J mice (aged 6–8 weeks), designated as oocyte donors, were fed either a high fat diet (HFD; 60% kcal from fat) or a normal diet (ND; 10% kcal from fat) for 10 weeks. Body weight was measured weekly to monitor development of diet-induced obesity. **(B)** A parallel cohort of CD-1 female mice, which served as embryo transfer surrogate recipients, was subjected to the same diet conditions (ND or HFD) for 10 weeks. These mice also exhibited significant weight gain under HFD conditions (p = 0.0001, α = 0.05), consistent with an obesity phenotype. In both panels, data are presented as mean ± SD, with n = 28 (GAM) and n = 40 (SUR) biological replicates per strain. Statistical significance was assessed using one-way ANOVA with Bonferroni post hoc correction. These two diet-conditioned maternal cohorts were used to generate experimental groups via IVF and embryo transfer, as outlined in Figure 1A-B of the main text.

 https://doi.org/10.3390/cells14151201

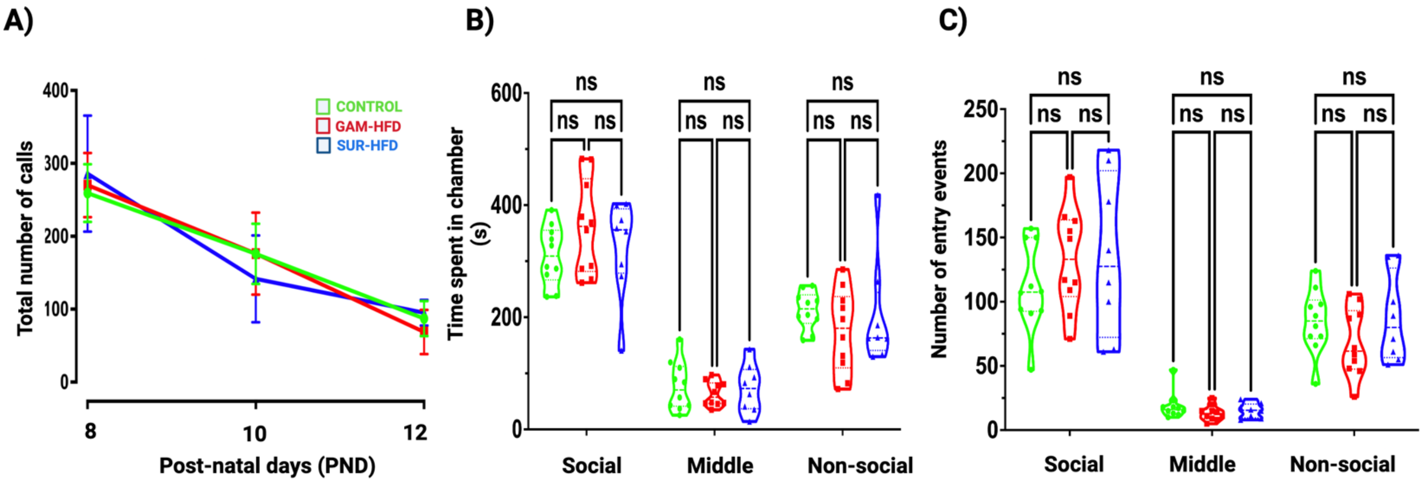

Figure S2. Female offspring from all embryo transfer groups did not exhibit ASD-like behavioral phenotypes. Behavioral assays were performed on female offspring at postnatal day (PND) 8, 10, 12 and 25 from each of the three embryo transfer groups: CONTROL, GAM-HFD, and SUR-HFD (Figure 1A-C). **(A)** The USV test was performed to assess communication at PND 8, 10, and 12. The data are expressed as the total number of calls made in the duration of the test. No significant differences were observed between groups at any time point. Social interaction was evaluated at PND 25 using the three-chamber test. No differences were detected in time spent investigating the novel mouse ("social" chamber) versus the empty ("non-social") chamber or between them ("middle" chamber) **(B)**, or in the number of entry events within those chambers **(C)**, suggesting a lack of social deficits. Data represent mean ± SD. Sample sizes were GAM-HFD (n = 10), and SUR-HFD (n = 8), and CONTROL (n = 10). Statistical analysis was performed using one-way or two-way ANOVA followed by Turkey's post hoc test; ns = not significant. Based on the absence of behavioral abnormalities in female offspring across groups, molecular analyses were performed exclusively on male offspring to focus on ASD-relevant phenotypes.

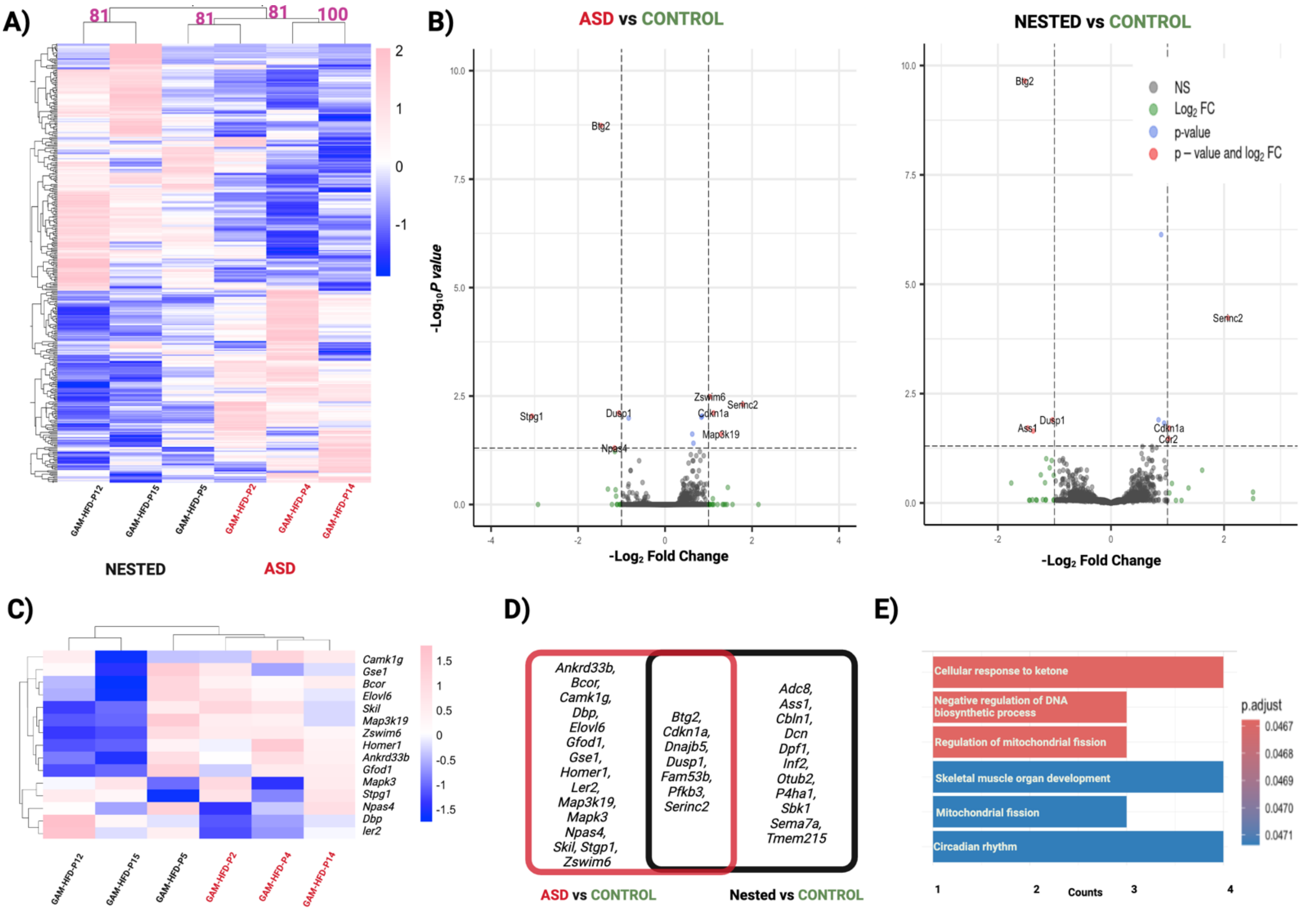

Figure S3. Hierarchical clustering, differential gene expression, and gene set enrichment analyses of cortical transcriptomes from male offspring. (A) Unsupervised hierarchical clustering based on global cortical transcriptomes from male offspring at PND 41 using Euclidean distance and complete linkage. Groups include GAM-HFD offspring stratified by ASD-like behavior (ASD and NESTED) and CONTROL mice. Distinct transcriptional profiles emerged, with the NESTED group exhibiting partial overlap with ASD. Red numbers at tree nodes indicate bootstrap support values from 10,000 replicates (values ≥ 0.80 denote statistically stable branches). **(B)** Volcano plots show differentially expressed genes (DEGs) between ASD vs CONTROL (left) and NESTED vs CONTROL (right). Genes were considered significant at $|\log_2 \text{fold change}| \geq 1$ and FDR-adjusted $p < 0.05$. **(C)** Heatmap of genes with unadjusted $p < 0.1$ from the ASD vs NESTED comparison, to highlight potentially biologically relevant transcriptional trends. **(D)** Venn diagram of DEGs (FDR < 0.1) comparing ASD vs CONTROL and NESTED vs CONTROL. Fifteen genes were unique to ASD mice. **(E)** Gene set enrichment analysis (GSEA) for the ASD vs CONTROL comparison, showing selected enriched pathways. Bar lengths indicate gene counts; color denotes FDR-adjusted p-values. RNA-Seq group sizes were ASD ($n = 3$), NESTED ($n = 3$), CONTROL ($n = 3$). Gene set analyses were based on the Molecular Signatures database MSigDB v 7.4 with significance threshold of FDR < 0.05.

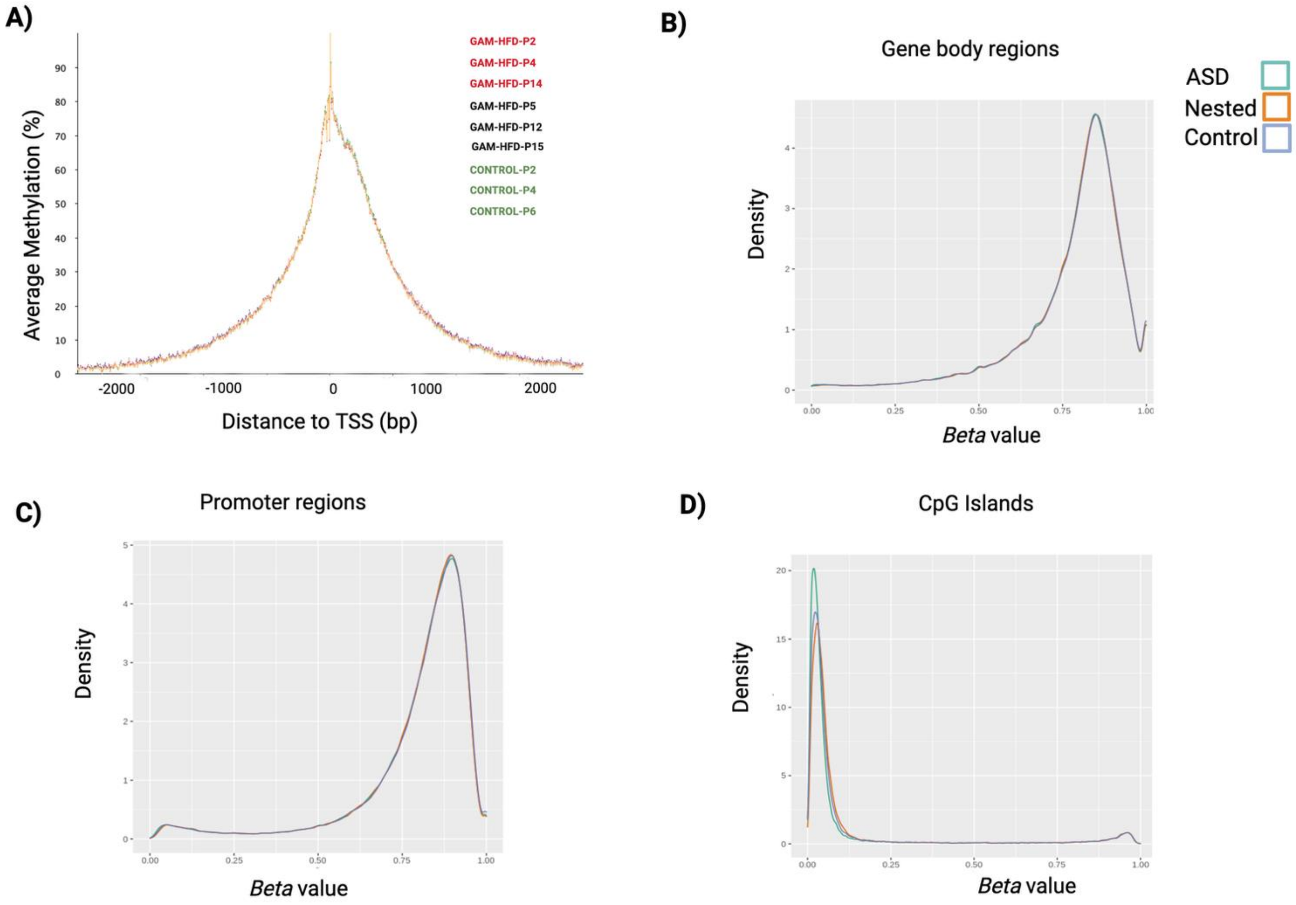

Figure S4. Global DNA methylation landscape across hippocampal genomes of male offspring. Whole-genome bisulfite sequencing (WGBS) was performed on hippocampal tissues collected at PND 41 from male offspring. **(A)** Average CpG methylation levels (in %) across the transcription start site (TSS), spanning ±2 kb. As expected, methylation levels sharply decline at the TSS, forming a distinct "V"-shaped pattern. This is a well-established epigenomic feature that generally reflects promoter accessibility and active transcription. **(B–D)** Density distributions of CpG methylation values (β-values) across three genomic feature classes: gene body regions (B), promoter regions defined as ±1 kb from the TSS (C), and CpG islands (D). The observed patterns align with canonical brain methylation architecture. Gene bodies are highly methylated, consistent with active transcription. Promoter regions display a broader range of methylation, capturing both active and repressed regulatory states. CpG islands are largely unmethylated, supporting their role in regulating gene expression at key loci. These consistent and expected methylation profiles reinforce the technical integrity of the WGBS data and suggest that the hippocampal epigenome in this model retains common organizational features despite exposure to pre-conceptional or gestational obesity. Group comparisons include CONTROL and GAM-HFD (ASD and NESTED) offspring. Each group includes three biological replicates. Statistical comparisons were made using the Kruskal–Wallis test with false discovery rate correction for multiple testing.

Table S1. In vitro fertilization (IVF) and embryo transfer summary across experimental groups.

Exp. group	IVF ID	No of oocyte donors used	No of oocytes inseminated	No of 2-cell embryos obtained	Surrogate ID and diet	No of embryos transferred	No of pups (males/females)
GAM-HFD	IVF 1	5	127	72	ND 1	12	9 (5/4)
					ND 2	12	3 (2/1)
					ND 3	12	7 (2/5)
					ND 4	0	0
					ND 5	12	10 (6/4)
					ND 6	12	10 (6/4)
					All	72	39 (21/18)
	IVF 2	5	125	65	ND 7	12	8 (2/6)
					ND 8	14	0
					ND 9	13	6 (3/3)
					HFD 1	13	7 (3/4)
					HFD 2	13	9 (3/6)
					All	65	30 (11/19)
	IVF 3	4	91	40	ND 10	13	8 (2/6)
					ND 11	13	9 (6/3)
					HFD 3	14	7 (4/3)
					All	40	24 (12/12)
GAM-ND	IVF 1	5	86	37	ND 12	12	7 (5/2)
					ND 13	12	0
					ND 14	13	8 (3/5)
					All	37	15 (8/7)
	IVF 2	5	102	50	ND 15	12	7 (4/3)
					ND 16	12	0
					HFD 4	13	8 (5/3)
					HFD 5	13	7 (3/4)
					All	50	22 (12/10)
	IVF 3	4	92	43	ND 17	13	6 (2/4)
					ND 18	14	7 (5/2)
					ND 19	16	7 (5/2)
					All	43	20 (12/8)

Abbreviations used: IVF, in vitro fertilization; GAM, gamete; ND, normal diet, HFD, high fat diet. Number and sex of pups was scored on the day of delivery. All females were confirmed as pregnant except for ND 13. Lack of pregnancy for ND 13 was likely because this female was not pseudopregnant. The lack of pups observed in the remaining females was likely due to immediate cannibalization after delivery.

Table S2. List of male offspring derived by IVF, embryo transfer and cross-fostering, and subjected to behavioral and molecular analyses.

Experimental group	IVF ID/ SUR ID	Pup ID	Foster litter ID	Behavioral phenotype	RNA-Seq and WGBS	Molecular analysis ID	Group
CONTROL	IVF-1	GAM-ND-IVF-1-P1	8	Unaffected			
	IVF-1	GAM-ND-IVF-1-P2	8	Unaffected	X	Control-P2	CONTROL
	IVF-1	GAM-ND-IVF-1-P3	9	Unaffected			
	IVF-1	GAM-ND-IVF-1-P4	9	Unaffected	X	Control-P4	CONTROL
	IVF-1	GAM-ND-IVF-1-P5	10	Unaffected			
	IVF-1	GAM-ND-IVF-1-P6	10	Unaffected	X	Control-P6	CONTROL
	IVF-2	GAM-ND-IVF-2-P17	12	Unaffected			
GAM-HFD	IVF-1	GAM-HFD-IVF-1-P2	1	Affected	X	GAM-HFD-P2	ASD
	IVF-1	GAM-HFD-IVF-1-P4	2	Affected	X	GAM-HFD-P4	ASD
	IVF-1	GAM-HFD-IVF-1-P5	2	Unaffected	X	GAM-HFD-P5	NESTED
	IVF-1	GAM-HFD-IVF-1-P12	3	Unaffected	X	GAM-HFD-P12	NESTED
	IVF-1	GAM-HFD-IVF-1-P14	6	Affected	X	GAM-HFD-P14	ASD
	IVF-1	GAM-HFD-IVF-1-P15	6	Unaffected	X	GAM-HFD-P15	NESTED
	IVF-3	GAM-HFD-IVF-3-P58	22	Affected			
SUR-HFD	SUR-HFD-9	SUR-HFD-9-P1	13	Unaffected			
	SUR-HFD-5	SUR-HFD-5-P1	14	Unaffected			
	SUR-HFD-5	SUR-HFD-5-P2	14	Unaffected			
	SUR-HFD-2	SUR-HFD-2-P2	15	Unaffected			
	SUR-HFD-2	SUR-HFD-2-P3	15	Unaffected			
	SUR-HFD-10	SUR-HFD-10-P1	16	Unaffected			
	SUR-HFD-10	SUR-HFD-10-P3	16	Unaffected			

Abbreviations used: IVF, in vitro fertilization; HFD, high fat diet; ND, normal diet; SUR, surrogate; GAM, gamete. Pups from the CONTROL group were derived from oocytes from ND-fed females and pregnancy carried by ND-fed surrogates. Pups from GAM-HFD group were derived from oocytes from HFD-fed females and pregnancy carried by ND-fed surrogates. Pups from SUR-HFD group were derived from oocytes from ND-fed females and pregnancy carried by HFD-fed surrogates.

Table S3. Functional enrichment analysis of the candidate genes associated with ASD phenotype.

Gene	Chr.	Gene Ontology (BP)	KEGG	MGI Enrichment	MGI Phenotype
Ankrd33b	15	DNA binding			
Bcor	X	Cellular component organization<br>DNA-templated transcription<br>System development	Polycom repressive complex	Histone deacetylase binding<br>Negative regulation of transcription by RNA polymerase II<br>Blastocyst hatching	**Abnormal developmental patterning**<br>Abnormal forebrain development<br>Abnormal gastrulation
Btg2	1	Protein methylation<br>Neuroblast proliferation<br>DNA-templated transcription<br>Response to stimulus<br>Programmed cell death	RNA degradation	Negative regulation of transcription by RNA polymerase II<br>Protein methylation<br>Associative learning<br>Response to mechanical stimulus<br>Central nervous system neuron development	**Behavior Neurological Hyperactivity**<br>Increased fasting circular glucose level
Camk1g	1	ATP binding<br>Response to stimulus<br>Signaling	Calcium/calmodulin-dependent protein kinase I	Signal transduction<br>Calcium and calmodulin dependent protein kinase complex	Abnormal dendrite morphology
Cdkn1a	17	Cell differentiation<br>Programmed cell death<br>Response to stimulus<br>System development	PI3K-Akt signaling pathway<br>Oxytocin signaling pathway	Regulation of cyclin-dependent protein serine threonine kinase activity<br>In utero embryonic development<br>Regulation of mitotic cell cycle	Abnormal bone marrow cell morphology/development<br>Abnormal cell cycle<br>Abnormal neural precursor proliferation<br>Abnormal response to CNS ischemic injury<br>Increase body weight<br>Increase brain weight

					Increase brown adipose tissue amount<br>Increase susceptibility to induced morbidity/mortality<br>Premature death
Dbp	7	Circadian rhythm<br>Rhythmic process	Environmental adaptation: Circadian rhythm<br>Transcription factor	DNA-binding transcription activator activity<br>RNA polymerase II-specific, Circadian rhythm	**Abnormal locomotor circadian rhythm**<br>**Shortened circadian behavior period**<br>**Decreased locomotor activity**
Dusp1	17	Cellular response to hormone stimulus<br>Intracellular signal transduction<br>MAP kinase tyrosine serine threonine phosphatase activity	MAPK signaling pathway<br>Serotonergic synapse<br>Parkinson disease	Endoderm formation<br>Chromatin remodeling	**Abnormal locomotor activation**<br>**Abnormal eating behavior**<br>Decrease abdominal fat pad weight<br>Decreased susceptibility to age-related obesity<br>Decrease susceptibility to diet-induce obesity
Elovl6	3	Long and very long-chain fatty acid biosynthetic process.<br>Fatty-acyl-CoA biosynthetic process	Biosynthesis of unsaturated fatty acids<br>Fatty acid elongation	Embryonic lethality, incomplete penetrance	**Decreased exploration in new environment**<br>Abnormal fatty acids level
Fam53b	7	Protein import into nucleus<br>Positive regulation of canonical Wnt signaling pathway<br>Regulation of canonical Wnt signaling pathway		Protein import into nucleus<br>Regulation of canonical Wnt signaling pathway	**Abnormal auditory brainstem response**<br>Increased cardiac stroke volume
Gfod1	13			Oxidoreductase activity<br>Nucleotide binding<br>Identical protein binding	**Abnormal locomotor behavior**
Gse1	8		Genetic information processing: Histone	Placenta development<br>I*n utero* embryonic development	Abnormal bone mineralization<br>Decreased body weight

			modification proteins HDAC complexes<br>BRAF-HDAC complex		Increased circulating total protein level
Homer1	13	G protein-coupled glutamate receptor signaling pathway<br>Regulation of postsynaptic neurotransmitter receptor activity<br>Dendritic spine maintenance<br>Behavioral response to cocaine	Environmental Information Processing: FoxO signaling pathway-2<br><br>Nervous system: Glutamatergic synapse	G protein-coupled glutamate receptor signaling pathway<br>Regulation of postsynaptic neurotransmitter receptor activity<br>Circadian rhythm<br>Behavioral response to cocaine	Abnormal nervous system physiology and enhanced behavioral response to cocaine<br>**Abnormal long-term spatial reference memory**<br>**Abnormal touch/ nociception**<br>**Impaired contextual conditioning behavior**<br>**Enhanced conditioned place preference behavior**
Mapk3	7	Cell differentiation<br>Programmed cell death<br>System development	EGFR tyrosine kinase inhibitor resistance<br>Central carbon metabolism in cancer<br>Pathways in neurodegeneration<br>Alzheimer disease<br>Age-RACE signaling pathway in diabetes complication<br>Insulin signaling pathway<br>Glutamatergic, Cholinergic and Serotonergic synapse<br>Long-term depression<br>Signaling pathways regulating pluripotency of stem cells	MAPK cascade<br>DNA-templated transcription<br>Protein phosphorylation<br>Apoptotic process<br>DNA damage response<br>Cell surface receptor signaling<br>Sensory perception of pain<br>Positive regulation of telomere maintenance<br>Response to lipopolysaccharide<br>Insulin-like growth factor receptor signaling pathway<br>Modulation of chemical synaptic transmission<br>Regulation of DNA-binding transcription factor activity<br>Stress-activated MAPK cascade<br>Bergmann glial cell differentiation	**Abnormal active/passive avoidance behavior**<br>Embryo phenotype<br>Decreased interleukin-1 beta secretion<br>Growth/size/body phenotype<br>**Increased locomotor activity**<br>Mortality/aging

Map3k19	1	DNA-dependent protein kinase activity	Protein kinase	MAPK cascade<br>Chromatin remodeling	
Npsa4	19	Cellular response to mineralocorticoid stimulus<br>Inhibitory postsynaptic potential.<br>Inhibitory synapse assembly<br>Short-term memory<br>Cellular response to ketone	Genetic information processing: Transcription factors	Cellular response to ketone<br>Cellular response to stress<br>Cellular response to corticosterone stimulus<br>Inhibitory synapse assembly<br>**Social behavior**<br>Regulation of synaptic plasticity<br>Short and long-term memory	**Abnormal behavior**<br>**Increased anxiety-related response**<br>Abnormal miniature excitatory postsynaptic currents<br>Neuron degeneration
Serinc2	4	phosphatidylserine metabolic process<br>phospholipid scramblase activity		Phosphatidylserine metabolic process	Impaired glucose tolerance
Skil	3	Cell differentiation<br>DNA-templated transcription<br>Programming cell death<br>Response to stimulus<br>System development		Negative regulation of transcription by RNA polymerase II<br>Blastocyst formation<br>Transforming growth factor beta receptor signaling pathway<br>Spermatogenesis<br>Extrinsic apoptotic signaling pathway via death domain receptors<br>Intrinsic apoptotic signaling pathways in response to DNA damage<br>Positive regulation of axonogenesis	Cachexia<br>Early cellular replicative senescence<br>Embryonic lethality before implantation, complete penetrance<br>Failure of zygote cell division<br>Immune system phenotype
Stpg1	4	Cellular component organization<br>Establishment of localization<br>Programmed cell death		Positive regulation of mitochondrial membrane permeability involved in apoptotic process	
Zswim6	13	GABAergic neuron differentiation	Signaling and cellular processes	Striatal medium spiny neuron differentiation	Abnormal cerebral cortex morphology

	Striatum development		Striatal medium spiny neuron differentiation.	**Abnormal grooming behavior**
	Subpallium development		Regulation of neuron migration	**Decreased anxiety-related response**
				**Increased stereotypic behavior**
				**Increased aggression**

Gene set enrichment analysis (GSEA) was performed with annotations for differentially expresses genes to understand their potential roles in behaviors associated with Autism Spectrum Disorders (ASD). Gene Ontology (GO), particularly Biological Process (BP), describes the biological process to which the gene products contributed. Additionally, the Kyoto Encyclopedia of Genes and Genomes (KEGG) and the Mouse Genome Informatics (MGI) database were used to identify specific biological contexts related to ASD with experimental evidence filter. The bolded terms highlight the key phenotypic behaviors impacted in ASD, including social interactions, communication, repetitive behaviors, and sensory processing resembling human ASD.
